# Development of a Flexible Sensor-Integrated Tissue Patch to Monitor Early Organ Rejection Processes Using Impedance Spectroscopy

**DOI:** 10.3390/bios14050253

**Published:** 2024-05-17

**Authors:** Peter Ertl, Tibor Wladimir, Drago Sticker, Patrick Schuller, Mario Rothbauer, Georg Wieselthaler, Martin Frauenlob

**Affiliations:** 1 Institute of Applied Synthetic Chemistry, Faculty of Technical Chemistry, Vienna University of Technology, Getreidemarkt 9/163, 1060 Vienna, Austria; 2 Karl Chiari Lab for Orthopaedic Biology, Department of Orthopedics and Trauma Surgery, Medical University of Vienna, Währinger Gürtel 18-22, 1090 Vienna, Austria; 3 Department of Surgery, Division of Cardiothoracic Surgery, University of California, San Francisco, CA 94143, USA

**Keywords:** environmental monitoring, nasal epithelial cells, nanotoxicology, lab-on-a-chip, impedance sensors, zinc oxide toxicity

## Abstract

Heart failure represents a primary cause of hospitalization and mortality in both developed and developing countries, often necessitating heart transplantation as the only viable recovery path. Despite advances in transplantation medicine, organ rejection remains a significant post-operative challenge, traditionally monitored through invasive endomyocardial biopsies (EMB). This study introduces a rapid prototyping approach to organ rejection monitoring via a sensor-integrated flexible patch, employing electrical impedance spectroscopy (EIS) for the non-invasive, continuous assessment of resistive and capacitive changes indicative of tissue rejection processes. Utilizing titanium-dioxide-coated electrodes for contactless impedance sensing, this method aims to mitigate the limitations associated with EMB, including procedural risks and the psychological burden on patients. The biosensor’s design features, including electrode passivation and three-dimensional microelectrode protrusions, facilitate effective monitoring of cardiac rejection by aligning with the heart’s curvature and responding to muscle contractions. Evaluation of sensor performance utilized SPICE simulations, scanning electron microscopy, and cyclic voltammetry, alongside experimental validation using chicken heart tissue to simulate healthy and rejected states. The study highlights the potential of EIS in reducing the need for invasive biopsy procedures and offering a promising avenue for early detection and monitoring of organ rejection, with implications for patient care and healthcare resource utilization.

## 1. Introduction

Heart failure is the largest cause of hospitalization and death in developed countries and one of the leading causes of disease burden in developing countries [1,2,3]. Besides congenital defects, reasons for heart failure can be derived from an unhealthy lifestyle, including stress, excess weight, smoking, or alcohol consumption [4,5]. Consequently, for many patients, heart transplantation is the only viable option for recovery, and therefore, since the early 1990s, the number of cardiac transplants worldwide has reached an annual rate of over 2000 surgical interventions per year [6]. Since the first human-to-human heart transplant operation was performed in 1967 by Christiaan Barnard at the Groote Schuur Hospital in Cape Town, the survival rate for heart transplant patients has improved over time, mainly due to improved survival in the early post-transplant period [7]. However, the first months following surgery are still crucial to the patient’s healing process. Despite all the efforts of modern medicine, heart graft rejection is still an important cause of death in patients with cardiac transplantation. One difficulty of successful therapy is that the patient usually remains asymptomatic until significant myocardial damage results in heart failure, and about one-fifth of the recipients die within a year of the operation [8,9]. Thereafter, the mortality rate stays constant, at about 4 percent a year for the next 18 years, so that only about 30 percent of patients can expect to be alive after 18 years [10]. The gold standard method of diagnosing and monitoring allograft rejections after heart transplantation is the endomyocardial biopsy (EMB), where myocardial tissue biopsies are surgically removed for histological examination. Here, the level of leucocyte infiltration into the removed cardiac tissue (e.g., none, low, medium, and high) is taken as an indicator of a potential risk of transplant rejection [11]. Although a routine procedure, endomyocardial biopsies are still associated with a risk of both procedural and long-term complications [12]. Particularly, the necessity of additional surgeries during the first posttransplant year and their frequency early after surgery have been questioned by the medical community [13,14,15]. Despite these concerns, heart transplant patients undergo repeated endomyocardial biopsy procedures during a prolonged period after surgery and are, therefore, at even higher risk of long-term complications from the biopsy. The main reason is that chronic organ rejection may take place within several days and weeks of hospitalization [16,17], making early detection of the onset of transplant rejection a medical necessity. Independent of the medical risk discussion and the considerable psychological strain put on patients, regular and close-meshed surgical procedures are also time- and cost-demanding on the part of health insurance and healthcare professionals [18]. 

**Figure 1:**
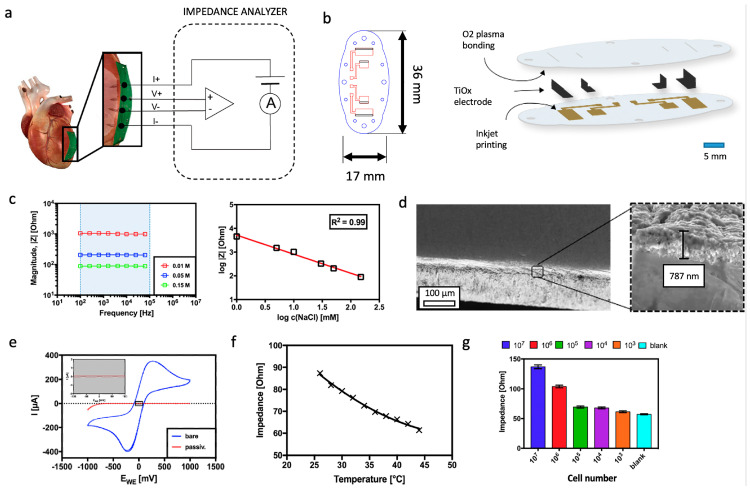
(**a**) Concept for the flexible tetra-polar setup for impedance spectroscopy analysis and tissue rejection monitoring using a smart implant. (**b**) Schematic overview and dimensions of the smart implant. (**c**) Influence of ionic strength and frequency on impedance spectra of the tetrapolar TiOx electrode setup. (**d**) Scanning electron micrographs of the oxide passivation layer after thermal oxidation of the electrodes. (**e**) Characterization of passivation quality of TiOx electrodes using cyclic voltammetry. (**f**) Influence of temperature (**g**) and cell density on impedance using tetra-polar measurement set-up of the smart implant.

To advance organ transplant rejection monitoring, we have developed a sensor-integrated flexible patch that can be sutured to any tissue type prior to implantation to continuously monitor resistive and capacitive changes that may occur during the complex tissue rejection process. The method is called electrical impedance spectroscopy, which is frequently applied for analysis of tissue and in vitro cell cultures [19,20,21,22,23,24] as well as heartbeat and respiration rate monitoring in sports medicine [25]. Furthermore, bioimpedance techniques are commonly used in quality control applications, among others, in fresh meat testing and fermentation analysis in the food and beverage industry [26]. More recent applications include stem cell differentiation monitoring, [27,28] cytotoxicity screening, [29,30] cell spreading [31], and immune cell activation [32]. In the present study, contactless impedance sensing using titanium-dioxide-coated (passivated) electrodes is applied as a label-free method to readily identify the onset and progression of cardiac rejection processes [19]. Here, the application of electrode passivation protocols using biocompatible, inert, and durable metal-oxide coatings is important to avoid electrode polarization events occurring at the electrode surface that may mask underlying cellular processes such as initial immune cell infiltration. Another key design feature of our biosensor concept is the integration of three-dimensional (3D) microelectrode protrusions capable of penetrating the muscle tissue within a flexible carrier substrate made of PDMS to match a) the heart’s curvature while simultaneously following muscle contraction events (see Figure 1a). In this study, the passivation layer consistency is evaluated using scanning electron microscopy as well as cyclic voltammetry, whereas frequency-dependent sensor performance is evaluated using SPICE simulations based on impedance spectra obtained from healthy and compromised “rejected” tissues. Sensor performance evaluation is conducted using fresh and freeze–thaw chicken hearts following repeated cycling processes to characterize cell membrane and tissue disruption events. Finally, sample-to-sample and device-to-device variations are studied by signal changes in resistance (real part) and reactance (imaginary part) at a frequency range between 1 Hz and 500 kHz by using healthy chicken heart tissue samples and monitoring the influence of enzymatic tissue digestion on them.

## 2. Materials and Methods

### 2.1. Electrode Passivation Using Thermal Oxidation of Titanium Electrodes

Thermal oxidation of 3 × 6 mm titanium sheets (thickness 0.25 mm) was performed under normal atmospheric conditions using a Skokan Edition 85 oven equipped with a Bentrup TC 66 compact controller (Bentrup, Ferwald, Germany) as described elsewhere [33]. First, samples were cleaned with acetone and ethanol in an ultrasonic bath for 25 min at room temperature and subsequently heated to 650 °C at a heating speed of 5 °C per minute. After 1 h, the oven was switched off, and the samples were cooled down to room temperature overnight. Evaluation of the surface modification was performed using scanning electrode microscopy (SEM) imaging at 5 kV in high vacuum mode (Zeiss, Supra40, Oberkochen, Germany). Passivation quality assessment of the metal oxide layer was next performed by employing cyclic voltammetry of the electroactive compound ferricyanide using a multichannel potentiostat (VMP3/P-01, Bio Logic, Seyssinet-Pariset, France) to confirm the absence of faradaic currents. Cyclic voltammetry measurements were carried out in a two-terminal configuration, using a passivated titanium wire as the working electrode and an external platinum wire as the reference/counter electrode. A scan rate of 16 mV/s was chosen, and the electric current was recorded in the absence and presence of 1 mM potassium hexacyanoferrate (II) trihydrate K_4_Fe(CN)_6_·3H_2_O and 1 mM potassium hexacyanoferrate (III) K3Fe(CN)6 dissolved in phosphate-buffered saline [34,35].

### 2.2. Biosensor Fabrication and Assembly

A sensor layout for a tetrapolar electrode setup containing a top and bottom layer was designed using AutoCAD (AutoDesk, Singapore). The layers were cut by a CNC laser cutter. Commercially available 250 µm thick clear PDMS sheets (MVQ Silicones, Weinheim, Germany) were silanized to achieve sufficient bonding of inkjet-printed circuits, as reported elsewhere [36]. In short, oxygen plasma-activated PDMS sheets were immersed for 60 min in a solution containing 5% (*v*/*v*) (3-mercaptopropyl)trimethoxysilane (MPTMS) diluted with tetrahydrofuran and 0.4% HCl. The PDMS sheets were sonicated in isopropyl alcohol for 5 min and then in ultrapure water for 5 min. Subsequently, substrates were dried under a stream of nitrogen.

For inkjet printing of conductive thin film electrodes, PDMS sheets were placed on a pre-heated silicon wafer, and the printer was set to a baseline height of 750 µm to adjust for the thickness of the PDMS sheet and the carrier wafer. Subsequently, the silver conductive tracks were patterned with a drop diameter of 70 μm on PDMS by a Dimatix inkjet printer (Lebanon, NH, USA) based on a CAD-designed layout featuring an inner electrode distance of 10 mm and a distance of 5 mm between the outer-to-inner electrode. After printing was finished, the PDMS substrates were annealed at 150 °C for 15 min. The passivation layer on one end of the electrode was removed using sandpaper. The electrodes were then fixed with the exposed end on the inkjet-printed tracks with conductive silver-containing epoxy glue and polymerized for 2 h at 75 °C. Subsequently, the top and bottom sensor layers were bonded using oxygen plasma at a chamber pressure of 700 mTorr for 30 s to form a tight seal.

### 2.3. Contactless Bioimpedance Sensing Using Passivated Titanium Electrodes

A commercial multichannel potentiostat (BioLogic, VMP3/P-01) was employed for impedance spectroscopy measurement controlled by the EC-Lab V11.02 software, enabling parallel measurement of currents down to 1 nA in the frequency range between 0.1 kHz and 1 MHz. In addition, to minimize the effects of stray capacitance, all measurements were corrected as described by [37].

### 2.4. Organ Sample Preparation

Commercially available animal tissue (grocery store, Austria) samples were examined electrochemically via impedance spectroscopy in order to obtain the electrical properties of biological tissue. Therefore, the sensor was inserted in the sample, which was then placed in a physiological saline bath, allowing continuous monitoring of structural changes within the tissue and alteration of electrical impedance during experiments. For the purpose of simulating pathological changes in tissue, two different experiments were planned and carried out. On the one hand, whole chicken heart tissue has been freeze–thawed several times and compared with the electrical impedance spectra of fresh and untreated samples. On the other hand, samples were treated with 1% Trypsin/EDTA in a physiological saline bath over 24 h at 37 °C in order to simulate the degradation of tissue.

### 2.5. Computational Simulation of Tissue Rejection Monitoring Using SPICE

The circuit analysis software package SPICE OPUS (v2.03) was used to investigate changes in bioimpedance due to changes in cell and tissue morphology. A two-dimensional image of tissue was sketched based on histological sections and converted into its equivalent circuit elements as a model of extracellular space expansion. The simulation was performed with a frequency sweep from 10 Hz to 1 MHz. For simplification, the simulations were performed in the absence of capacitive leakage at higher frequencies and polarization effects at frequencies below 1 kHz.

## 3. Results

### 3.1. Establishment and Characterization of the Tetrapolar Biosensor Setup Consisting of Oxygen-Passivated Titanium Electrodes

Prior to the biological evaluation of the implant device, electrochemical analysis using impedance spectroscopy and cyclic voltammetry was performed to evaluate the Ti-based electrodes and the consistency of the oxide passivation layer. As shown in Figure 1c, the proposed tetrapolar measurement setup shows linear responses in the presence of increasing sodium chloride concentrations (conductivity changes), which are independent of frequency (e.g., range between 100 Hz and 100 kHz). Importantly, in the presence of unphysiologically low (0.01 M) and high (0.15 M) sodium chloride concentrations, our bipolar measurement systems featured significant signal artifacts (e.g., highest impedance values), as shown in Figure S1a below 200 Hz and 1 kHz, respectively [38,39]. To further prevent the occurrence of unwanted electrode polarization effects, a tetrapolar measurement setup was implemented for all subsequent bioimpedance measurements in biological environments, thus adding to device complexity and biosensor integration. The change from a two-electrode to a four-electrode setup further eliminated unwanted stray capacitance of approximately 400 pF above 10 kHz, as shown in Figure S1b, while impedance recordings below 10 kHz remained unaffected using a previously described correction function [37]. 

Next, the quality of the oxidation layer was investigated in more detail using scanning electron microscopy (SEM) and cyclic voltammetry (CV) techniques to gain a better understanding of the biosensing surface characteristics that ultimately interface with the tissue structure. Here, several thin sections of titanium electrodes were prepared approximately 24 h after thermal oxidation (total of 4 h) and subjected to SEM and CV analyses. As seen in Figure 1d, a homogenous interfacial titanium dioxide layer with a thickness of 640 ± 180 nm (*n* = 6 individually fabricated biosensors) is found on top of the planar titanium electrode surfaces, indicating a complete oxidation procedure. Additional evaluation of the quality of the formed TiO_2_-layer is conducted using cyclic voltammetry to demonstrate the absence of any faradaic and direct electron transfer processes through the passivation layer. Here, pristine (not treated) and thermally oxidized Ti electrodes are immersed in a 1 mM ferricyanide solution, and current-voltage curves are recorded to detect defects in the passivation layer. While oxidation peak currents of approx. 350 mA are present in pristine titanium electrodes, [37] no faradaic currents are recorded following extended thermal oxidation at 650 °C for 1 h, as shown in Figure 1e. Another important factor to consider that may impact bioimpedance recordings is the varying tissue temperatures, which are known to take place during organ rejection [40]. Such post-transplant fevers induce natural variations within a physiological temperature range of approx. 4 °C (e.g., 36–40 °C) during measurements and therefore need to be accounted for. As indicated in Figure 1f, impedance signals need to be corrected for varying body temperatures in order to avoid an artificially lower tissue impedance due to a higher body temperature, which is estimated to decrease linearly by 2 Ohm/°C. To avoid batch-to-batch variations, in all subsequent experiments, the measurement temperature was held constant at 23 °C. Figure 1g confirms the sensitivity of the sensor to increasing Jurkat cells embedded in a hydrogel in the range of 10 Hz to 500 kHz with an impedance of 57.22 ± 0.78 Ohm for blank hydrogel and an impedance increase to 136.92 ± 3.08 Ohm for a simplistic dense tissue model (cell concentration of 10^7^ cells per mL hydrogel; *n* = 3 biological replicates).

**Figure 2:**
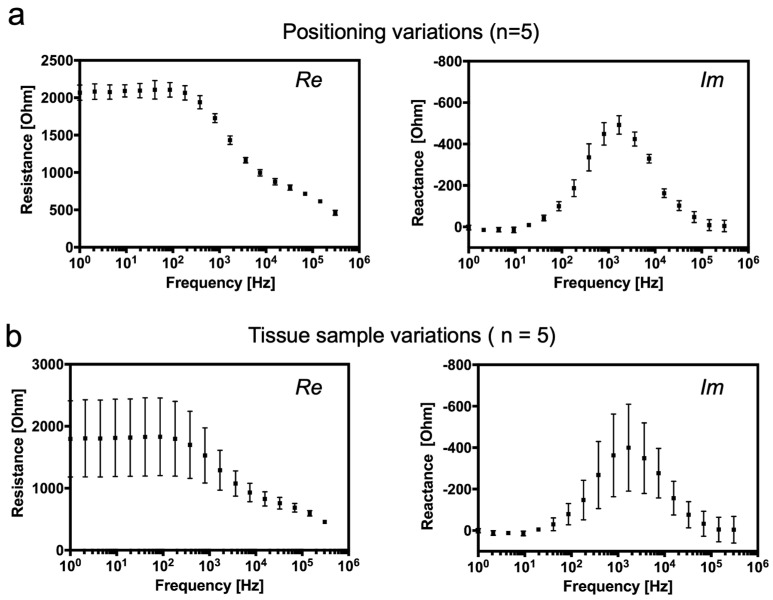
(**a**) Variation of impedance signal within the same tissue sample: (Re) real and (Im) imaginary impedance spectra of a chicken heart sample measured on various sensor positions in tissue (*n* = 5 technical replicates). (**b**) Variation of (Re) real and imaginary (Im) impedance between various chicken heart samples. (*n* = 5 biological replicates).

To evaluate whether the position of the sensor in tissue has an influence on the impedance results, the exact same tissue sample was measured using the same electrode setup several times in different positions. As shown in Figure 2a, there is just a negligible standard deviation in both real and imaginary impedance spectra of a chicken heart sample measured on various sensor positions. This may have been caused by a slight variation in heart tissue structure and by the local tissue damage caused by the electrode itself. Although a slight deviation was expected due to a variation in size and age of the samples, a surprisingly high deviation was found between chicken heart tissue samples with almost identical properties in both real and imaginary parts of the impedance signal, as shown in Figure 2b, respectively. Despite the high deviation, a characteristic Ø dispersion is still apparent. The resistance of biological tissue is mostly influenced by the extracellular passive electrical properties at lower frequencies, and therefore, at frequencies below 1 kHz, there is a high deviation of resistance, whereas the imaginary part is predominantly affected by the cell membrane, and gap junction reactance is highly sensitive at about 1 kHz.

Since the thermal oxidation of titanium electrodes, inkjet printing of leads (see example in Figure S2), and subsequent device assembly have been manually conducted in an academic setting, high sample-to-sample variations of our bioelectronic devices during the chicken heart experiments that we noticed with our academic prototypes can potentially be avoided when employing automated industrial manufacturing strategies such as CNC milling or punching machines that have a high control of structuring precision.

### 3.2. Computational and Experimental Estimation of Impedance Changes Caused by Declining Tissue Integrity

**Figure 3:**
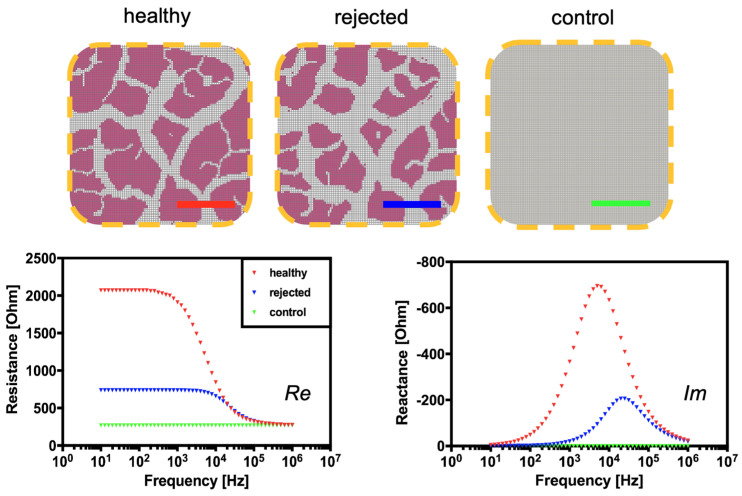
SPICE simulations and resulting impedance spectra of healthy and compromised “rejected” tissue after rejection in comparison to saline solution after the freeze–thaw cycling process for cell and tissue disruption.

To provide a theoretical foundation for the proposed passivated-electrode impedance monitoring concept for tissue rejections, SPICE computer simulations are performed. Figure 3 (left graph) shows the influence of tissue architecture on electrical impedance over the frequency range from 100 Hz to 100 kHz in the presence of a healthy and compromised biological sample. Changes in tissue integrity are apparent at characteristic frequencies between 100 Hz and 100 kHz in the real part of the electrical impedance, whereas changes in the extracellular space are visible below 100 Hz. Since tissue rejections are known to cause changes in extracellular edema, the results of the SPICE simulation indicate that both the resistive (real part) and the capacitive (imaginary part) electrical properties will significantly shift during tissue degradation processes. Results of the simulation show that at a frequency of 1 kHz, the loss of cell and tissue integrity and disruption of cellular membranes will induce a decrease in reactance.

**Figure 4:**
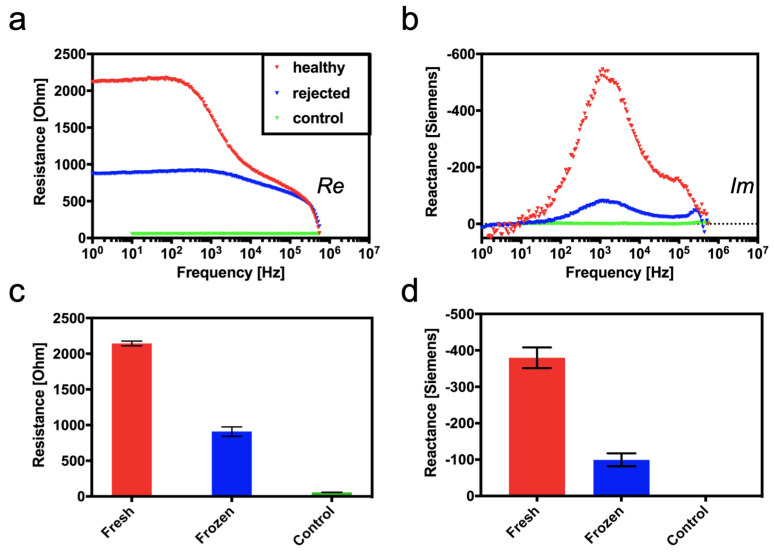
Relationship between morphological integrity of chicken heart tissue and impedance: (**a**) Real and (**b**) imaginary spectra of measured electrical impedance for fresh and treated tissue samples. Graphs (**c**) and (**d**) show an average of resistance and reactance of measured freeze-treated samples at characteristic frequencies of 100 Hz and 5 kHz, respectively. (*n* = 4 biological replicates).

To confirm the above computational results in an experimental setting, commercially available chicken hearts are used as a tissue degradation model in the next set of experiments. Here, a simplified heart tissue degradation is emulated by performing a controlled and repeated freezing–thawing cycle on chicken hearts to disrupt cell membranes and induce an increasing loss of tissue integrity. It is important to note that the effects of freezing–thawing processes on meat and muscle tissue are well-known in food sciences and food engineering [41]. Results seen in Figure 4a–d correlate well with the previously obtained simulation data, where physical tissue decomposition based on freezing and sequential thawing resulted in a resistance decrease from 2135.7 ± 9.9 Ohm to 881.9 ± 33.9 Ohm at a frequency of 10 Hz. A similar decrease, thus characteristic dispersion behavior for intact and compromised tissue, is observable between 10 Hz and 300 kHz. In the frequency range below 100 Hz; however, the impedance sensing device is most sensitive to the resistive changes of tissue decomposition. At frequencies above 100 Hz, the measurement sensitivity seems to be declining, resulting in no discernible signal differences above 300 kHz between intact and compromised heart tissues. Interestingly, a significant decrease in reactance is observed in the frequency range between 100 Hz and 300 kHz, with a maximum sensitivity at 1.1 kHz. In other words, a reactance drop from −518.3± 11.2 Ohm to −45.9 ± 3.7 Ohm occurred as a result of simple membrane disruption and loss of tissue integrity during organ degradation. In turn, as predicted by the SPICE simulations, the addition of buffered saline solution did not result in any characteristic impedance dispersions, resulting in a flat line over the measured frequency range for both real and imaginary parts of the impedance.

### 3.3. Continuous Monitoring of Structural Changes in Tissue Integrity Using a Flexible Sensor-Integrated Tissue Patch

**Figure 5:**
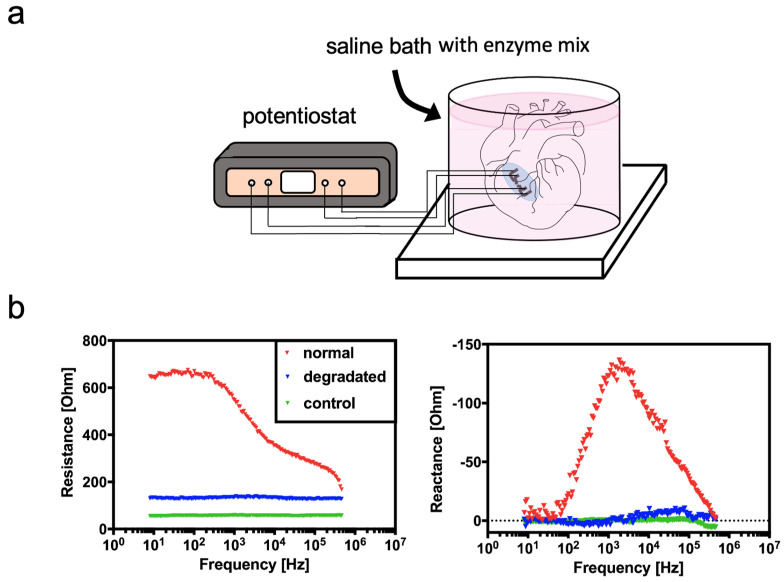
(**a**) Schematic of the heart-enzymatic heart-degradation setup for the sensor-integrated tissue patch. (**b**) Influence of enzymatic tissue digestion on smart implant performance for resistance (real part) and reactance (imaginary part) at a frequency range between 1 Hz and 500 kHz.

As a final proof-of-principle, chicken heart samples were subjected to enzymatic digestion for 24 h using a sterile trypsin/EDTA solution containing NaCl, as depicted in Figure 5a,b. Impedance data show the impact of enzymatic degradation on the electric properties of chicken heart samples, with a decrease in resistance over the whole frequency range from 10 Hz to 500 kHz, with the highest amplitude around 100 Hz. Even at a frequency of 500 kHz, a difference in resistance between non-compromised and enzymatically digested tissue is detectable. For the reaction, the highest sensitivity is found at 1.1 kHz in the presence of fresh tissue. However, a nearly complete loss in capacitive cell membranes is readily detectable up to 500 kHz, which stands in stark contrast to results obtained with our freezing protocol, where only a small peak of 70 Ohms remained in the reactance, indicating total tissue degradation and decomposition in comparison to the cellular effects shown in the previous freezing experiments.

**Figure 6:**
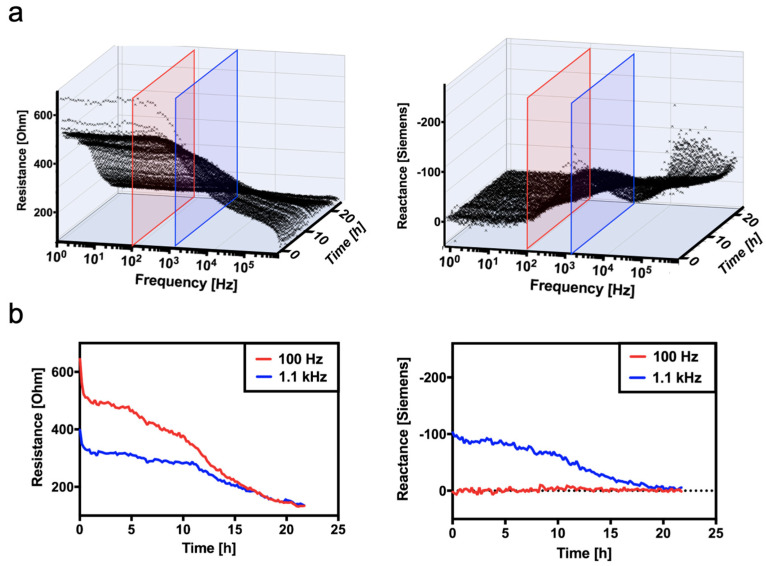
(**a**) Time-resolved impedance spectra and (**b**) the comparison of impedance-time curves of enzymatically degraded chicken heart tissue between the frequencies of 100 Hz and 1.1 kHz.

To better mimic tissue degradation, enzymatic tissue decomposition is next monitored in a time-resolved manner to monitor continuous changes in tissue composition and structure as disruption of cell membranes and the enlargement of extracellular tissue progress over time. Results in Figure 6a,b show three-dimensional impedance spectra (resistance and reactance) over the entire frequency range and measured time trace. Additionally, a simplified reactance trace follows the gradually degrading tissue samples over time. Interestingly, the time-dependent loss of resistance is detectable with the highest sensitivity at a frequency of 100 Hz, where impedance values gradually decline from 670.8 Ohm to 133.3 Ohm, compared to a frequency of 1.1 kHz (blue curve). The decrease in resistance values can be attributed to changes in tissue integrity and increasing extracellular space, which allows the flow of ions within the AC electric field more easily over time. It is also important to highlight that reactance measurements are more sensitive at higher frequencies, such as 1.1 kHz, where the reactance gradually decreased over 24 h from −127.4 Ohms to −3.3 Ohms (e.g., measurements at 100 Hz result in no sensor response over time).

## 4. Conclusions

Time-resolved and continuous monitoring of transplants is vital for the early detection of rejection events leading to organ rejection and implant failure. During organ rejection, several cellular and humoral processes take place over a period of several weeks and months, including inflammatory infiltration of immune cells, cardiac allograft vasculopathy, and cytomegalovirus infection [11]. Early detection of structural changes in tissue can, therefore, increase the rate of implant success since early drug administration can reduce morbidity resulting from implant failure. For instance, Basiliximab and Daclizumabis are frequently used as drugs to counteract the immune response during cellular rejection. However, these therapeutic approaches must be administered as soon as possible. Here, a flexible sensor-integrated tissue patch that can be sutured to any organ transplant could give vital information on the health status of the transplanted tissue, thus providing good indications for the need for clinical intervention. Although our current proof-of-concept study involved ex vivo, in vitro, and in silico approaches to demonstrate sensors, further investigations need to be performed in the future to demonstrate the feasibility of the flexible biosensor prototypes in preclinical studies on heart rejection using in vivo models. Of great importance will be material biocompatibility and the effect of beating cardiac tissue on impedance read-outs, which are both out of the scope of the current pilot study to develop and characterize sensor prototypes. Furthermore, not only in vivo sensor performance but also approaches to adapt the current sensors for wireless data transfer will improve the utility of the current prototypes in actual monitoring setups.

Although no animal testing (due to ethics restrictions at TU Wien) has been conducted in this study to confirm and validate the functionality of our sensor-integrated tissue patch, we believe that the basic operating principle of the proposed technology platform has been demonstrated. We envision, however, the additional integration of RFID technology for wireless communication and energy transfer between the sensing device and the sensing unit to eliminate the reliance on electrical wiring in a clinical setting. Any technological advancement in the area of transplant rejection monitoring needs to overcome frequent tissue movements, temperature variations, and device stability issues to ensure secure and reliable operation for months or years.

## Data Availability

Data will be provided upon reasonable request.

## Supplementary Materials

The following supporting information can be downloaded at: https://www.mdpi.com/article/10.3390/bios14050253/s1, Figure S1: Comparison of sensor response to sodium chloride solutions in the range of 10–150 mM for (a) impedance and (b) susceptance (X) of bipolar (top) versus tetrapolar (bottom) electrode set-ups; Figure S2: Example for rapid prototyped electrode leads on functionalized PDMS silicone substrates prior to TiOx attachment and back-end processing.
