# Supplementary material for: Development of a Flexible Sensor-Integrated Tissue Patch to Monitor Early Organ Rejection Processes Using Impedance Spectroscopy
Source: Biosensors (Basel). 2024 May 17;14(5):253. doi: 10.3390/bios14050253 (PMC11118035; doi:10.3390/bios14050253)
Supplement: Supplementary file 1 [file biosensors-14-00253-s001.zip › biosensors-3003117-supplementary.pdf]

biosensors

MDPI

Supplementary Materials

Development of a flexible sensor-integrated tissue patch to monitor early organ rejection processes using impedance spectroscopy

Peter Ertl [1&], Tibor Wladimir [1&], Drago Sticker [1&], Patrick Schuller [1], Mario Rothbauer [1,2,*], Gregor Wieselthaler [3] and Martin Frauenlob[1,*]

[1] Institute of Applied Synthetic Chemistry, Faculty of Technical Chemistry, Vienna University of Technology, Getreidemarkt 9/163, 1060 Vienna, Austria
[2] Karl Chiari Lab for Orthopaedic Biology, Department of Orthopedics and Trauma Surgery, Medical University of Vienna, Währinger Gürtel 18-22, 1090 Vienna, Austria
[3] University of California, Department of Surgery, Division of Cardiothoracic Surgery, San Francisco, CA
* Correspondence: martin.frauenlob@tuwien.ac.at; mario.rothbauer@tuwien.ac.at
& Authors contributed equally

Content: Figure S1, Figure S2

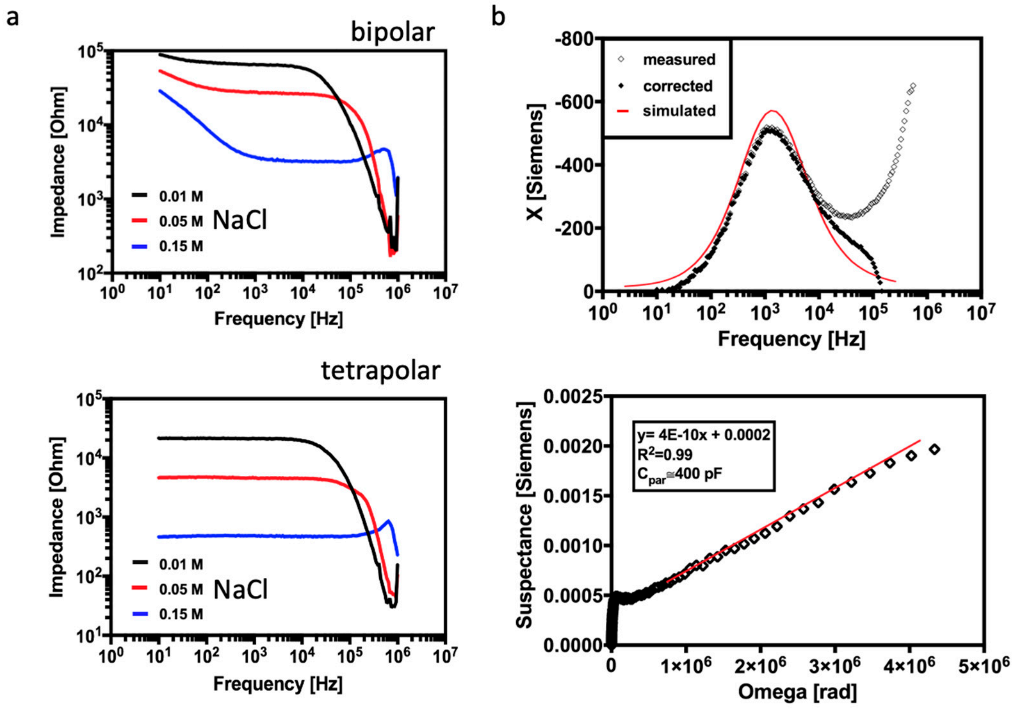

Figure S1. Comparison of sensor response to sodium chloride solutions in the range of 0.01-0.15 M for (a) impedance and (b) susceptance (X) of bipolar (top) versus tetrapolar (bottom) electrode setups .

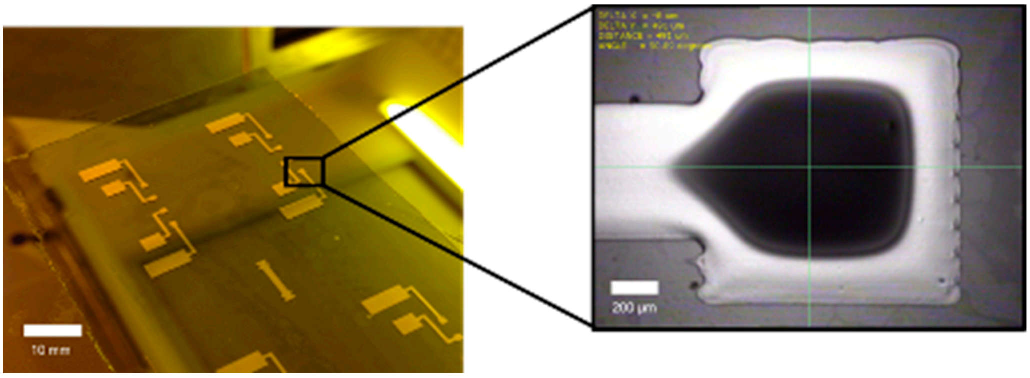

Figure S2. Example for rapid prototyped electrode leads on functionalized PDMS silicone substrates prior to TiOx attachment and back-end processing.

Disclaimer/Publisher's Note: The statements, opinions and data contained in all publications are solely those of the individual author(s) and contributor(s) and not of MDPI and/or the editor(s). MDPI and/or the editor(s) disclaim responsibility for any injury to people or property resulting from any ideas, methods, instructions or products referred to in the content.
